# Do patients with schizophrenia use prosody to encode contrastive discourse status?

**DOI:** 10.3389/fpsyg.2014.00755

**Published:** 2014-07-18

**Authors:** Amandine Michelas, Catherine Faget, Cristel Portes, Anne-Sophie Lienhart, Laurent Boyer, Christophe Lançon, Maud Champagne-Lavau

**Affiliations:** ^1^CNRS, LPL, UMR 7309, Aix-Marseille UniversitéAix-en-Provence, France; ^2^EA 3279 - Public Health, Chronic Diseases and Quality of Life - Research Unit, Aix-Marseille UniversitéMarseille, France; ^3^Department of Psychiatry, Sainte-Marguerite University HospitalMarseille, France

**Keywords:** prosodic phrasing, contrastive discourse status, theory of mind, social interaction, attribution of knowledge, schizophrenia, French

## Abstract

Patients with schizophrenia (SZ) often display social cognition disorders, including Theory of Mind (ToM) impairments and communication disruptions. Thought language disorders appear to be primarily a disruption of pragmatics, SZ can also experience difficulties at other linguistic levels including the prosodic one. Here, using an interactive paradigm, we showed that SZ individuals did not use prosodic phrasing to encode the contrastive status of discourse referents in French. We used a semi-spontaneous task to elicit noun-adjective pairs in which the noun in the second noun-adjective fragment was identical to the noun in the first fragment (e.g., BONBONS marron “brown candies” vs. BONBONS violets “purple candies”) or could contrast with it (e.g., BOUGIES violettes “purple candles” vs. BONBONS violets “purple candies”). We found that healthy controls parsed the target noun in the second noun-adjective fragment separately from the color adjective, to warn their interlocutor that this noun constituted a contrastive entity (e.g., BOUGIES violettes followed by [BONBONS] [violets]) compared to when it referred to the same object as in the first fragment (e.g., BONBONS marron followed by [BONBONS violets]). On the contrary, SZ individuals did not use prosodic phrasing to encode contrastive status of target nouns. In addition, SZ's difficulties to use prosody of contrast were correlated to their score in a classical ToM task (i.e., the hinting task). Taken together, our data provide evidence that SZ patients exhibit difficulties to prosodically encode discourse statuses and sketch a potential relationship between ToM and the use of linguistic prosody.

## Introduction

The term *prosody* refers to variations of supra-segmental features of speech, including pitch, duration and intensity. It serves important communication functions, either at the affective level—*emotional prosody* is known to convey emotions such as happiness, anger, fear, which clarify the emotional content of utterances—or at the linguistic level—*linguistic prosody* affects the meaning of what is being said. Among its linguistic functions, prosody reflects the way in which speakers structure information according to the knowledge they attribute to their interlocutor. We focus here on the linguistic function of prosody as it conveys what is part of the background (i.e., already given or previously-mentioned in the immediate discourse context) and what is part of a contrastive focus (i.e., the constituent of the message selected by the speaker to be underlined as opposed to another constituent in the immediate discourse context; Selkirk, 1984). For instance, in the following sentence, *Tu dois passer entre les BOUGIES violettes et les BONBONS violets* “You have to pass between the purple candles and the purple candies,” the noun *BONBONS* conveys the contrastive information relative to the noun *BOUGIES* as it has a contrastive focus discourse status. Conversely, the color adjective *violets* in “*BONBONS violets*” serves as a context since it is already mentioned in “*BOUGIES violettes*” (i.e., it has a given discourse status). Early work on languages such as English and Dutch provides evidence for a tendency to accent contrastive information and to refrain from accenting given information (Halliday, 1967; Chafe, 1976; Clark and Haviland, 1977; Cruttenden, 1986; Prince, 1986). More recent work focusing on stress-accent languages such as English shows that the correspondence between contrastive discourse status and the speaker's intonational expression of prominences requires pinpointing not just the presence/absence of accent on the words but also the particular accent type assigned (Pierrehumbert, 1980; Beckman and Pierrehumbert, 1986; Pierrehumbert and Hirschberg, 1990). In contrast, French does not use pitch accent types to encode contrastive status of words. Rather, it mainly relies on prosodic phrasing, or the grouping of words into prosodic units of different sizes (Féry, 2001; Dohen and Loevenbruck, 2004; Beyssade et al., 2009; Chen and Destruel, 2010). For instance, using a paradigm in which participants had to correct sentences produced by a fictitious interlocutor (a prompt), Dohen and Loevenbruck (2004) show that participants produced utterances with the verb phrase and the object noun phrase grouped together in the same prosodic constituent (the Accentual Phrase or AP) in contexts in which no element of the utterance contrasted with a previously-mentioned discourse element (e.g., *[Les loups]^AP^ [suivaient Mariloup]^AP^* “The wolves were following Mariloup”). However, they produced the element that contrasted with a previously mispronounced element in a separate AP when they knew that the prompt conveyed false-belief about this element (e.g., *[Les loups]^AP^ [suivaient]^AP^ [Mariloup]^AP^* when “Mariloup” was mispronounced).

Pre-boundary lengthening and pitch contours have been found to be the two main correlates of AP prosodic phrasing in French (see Pasdeloup, 1990; Jun and Fougeron, 2000, 2002; Astésano, 2001; Welby, 2006; Michelas and D'Imperio, 2012, *among others*). Specifically, the last full syllable of the AP has been shown to exhibit longer duration than unaccented syllables within an AP. In addition, APs in a non-final position within the utterance are typically characterized by a rising f0 contour aligned with the last full syllable of the AP, resulting in higher f0 values associated with AP-final syllables than with non-AP-final syllables. Note that due to the fact that accent always affects the last full syllable of the Accentual Phrase in French (see for example Jun and Fougeron, 1995, 2000, 2002; Welby, 2006; Michelas and D'Imperio, 2012), the last pitch accent of the AP “Mariloup” coincides with the boundary tone cuing the intonational phrase boundary (IP; *[[Les loups]^AP^ [suivaient]^AP^ [Mariloup]^AP^]^IP^*). In this example it is thus impossible to separate out the acoustic properties of the pitch accent from those of the boundary tone. However, one could argue that the same relationship between contrastive discourse status and the Accentual-Phrase right boundary could be found in a non IP-final position in French in a context in which the acoustic properties of the pitch accent are not superimposed with the acoustic properties of the boundary tone. Note also that prosodic cues such as the presence of an initial accent which is associated to the left edge of the AP could also mark the phrasing of the word “Mariloup” separately from the rest of the utterance (see for instance Beyssade et al., 2009; German and D'Imperio, 2010; D'Imperio et al., 2012).

Individuals with schizophrenia (SZ) experience deficits in social cognition, resulting in theory of mind (ToM) impairments and communication disorders (Brüne, 2005; Sprong et al., 2007; Green et al., 2008). ToM refers to the ability to form representations of other people's mental states (e.g., intentions, beliefs, and shared knowledge) and to use these representations to understand, predict, and judge their statements and behaviors (Premack and Woodruff, 1978; Baron-Cohen et al., 1999). Impairments of this ability have been investigated in SZ in situations involving natural communication (McCabe et al., 2004; Champagne-Lavau et al., 2009) as well as in tasks assessing first order false beliefs (such as *Mary believes it's raining*) and second order false beliefs (such as *Mary believes Paul thinks it's raining;* see for instance Mazza et al., 2001; Brüne, 2005; Bora et al., 2007; Champagne-Lavau and Stip, 2010), irony comprehension (Leitman et al., 2006; Kosmidis et al., 2008; Kern et al., 2009; Sparks et al., 2010; Champagne-Lavau et al., 2012), hinting comprehension (Corcoran et al., 1995; Janssen et al., 2003; Greig et al., 2004; Bora et al., 2006) and in picture-sequencing tasks (Langdon et al., 2006; Brüne et al., 2007). Turning to the field of communication disorders, SZ's language impairments appear to be primarily a disruption of pragmatics. However, SZ could also involve disruption at other linguistic levels including the phonetic one. For instance, a limited number of studies have addressed the question of how prosodic features are produced in SZ speech. In the literature, the prosody of SZ patients has been mainly described as “monotononous” or “abnormal” due to a reduction in the global pitch range resulting in flat intonation (see Rieber and Vetter, 1994 for a review of studies about flat intonation in SZ speech). Clemmer (1980) also found that SZ speech is also characterized by more pauses and hesitations than normal speech, though this may be at least partly the result of difficulties at the semantic or pragmatic level rather than a specifically prosodic impairment. Finally, other studies show that not only the production but also the comprehension of prosody as an expression of emotion is impaired (McCann and Peppé, 2003; Hoekert et al., 2007 for reviews). Therefore, this is the first investigation of how SZ individuals use prosody to encode contrastive status of discourse referents in French during a social interaction.

The use of prosody in the speech of individuals with social cognition disorders has also been addressed in autism. First, Chevallier et al. (2011) tested whether children with a high-functioning Autism Spectrum Disorder (ASD) vs. healthy control participants were able to use emotional prosodic cues to recognize the speaker's emotional state that relies on ToM. Although the results show that people with autism have subtle difficulties dealing with vocal cues, participants with ASD were not specifically impaired in conditions requiring higher order ToM skills, such as expressions of speakers' attitudes toward emotions attributed to others. More interestingly for our purpose, DePape et al. (2012) investigated the use of linguistic (rather than emotional) prosody in the speech of adults with ASD. In this experiment, participants were asked to answer questions inducing different samples of the same word (e.g., bed) either in a given context (e.g., “Who is painting the BED? The rabbit is painting the BED”) or in a focused context (e.g., “What is the rabbit painting? The rabbit is painting the BED”). They showed that English-speaking adults with ASD did not prosodically encode given vs. focus discourse status as typical English-speaking adults did. The authors suggest that this impairment was due to the level of language functioning of participants assessed by the standardized Peabody picture vocabulary test-III (Dunn and Dunn, 1997). This result established a correlation between the use of prosody to mark discourse status with a pathology affecting language abilities. However, since language functioning was measured with a vocabulary test, the authors did not address the question of the relationship between the participant's social disorders (and specifically their ToM difficulties) and their difficulties in using linguistic prosody.

Here we reported detailed acoustic analyses on the way individuals with SZ use linguistic prosody to encode contrastive status of discourse referents. We used a semi-spontaneous task during which participants were interacting with a conversational partner and had to produce pairs of noun-adjective pairs in which the noun in the second noun-adjective fragment was identical to the noun in the first fragment (e.g., *BONBONS marron* “brown candies” vs. *BONBONS violets* “purple candies”) or could contrast with it (e.g., *BOUGIES violettes* “purple candles” vs. *BONBONS violets* “purple candies”). Our goal was to compare the use of prosodic phrasing to encode the contrastive status of referents in healthy control participants (HC) and in SZ participants. Furthermore, as a secondary goal of our paper, we also examined whether the use of prosody of contrast could reflect SZ patients' difficulties in attributing knowledge to the person with whom they were interacting. It is important to notice that the prosodic encoding of discourse status appears to involve ToM. According to Clark (1996), all kinds of interactions consist in a permanent exercise of mindreading during which both the speaker and the listener carefully track their addressee's mental states throughout conversations. In the present study, we used a task-oriented dialog involving verbal cooperation between two participants, each holding a map, one with a route mapped out on it and the other without. The director had to tell the follower how to chart the route onto the follower's map as accurately as possible. To do so, he/she had to produce discourse contrasts. One of the striking features to encode discourse contrasts is the use of prosody. Thus, knowing whether individuals suffering from a pathology affecting social cognition have difficulties in using prosody to express pragmatic contrasts in a conversational situation, and understanding the nature of such a deficit, would be valuable in understanding the nature of the pathology.

Thus, our main goal was to compare the use of prosodic phrasing to encode the contrastive status of referents in healthy control participants (HC) and in SZ participants. As a secondary goal of the paper, we also examined whether this use could reflect SZ patients' difficulties in attributing knowledge to the person with whom they were interacting. To do so, we collected semi-spontaneous speech samples in a task-oriented dialog involving verbal cooperation between two participants.

## Methods

### Participants

We tested 10 SZ patients and 10 HC participants, matched for age and years of education. All SZ participants were outpatients recruited from the Department of Psychiatry of the Hôpital Sainte Marguerite in Marseille. Inclusion criteria were a DSM-IV-TR diagnosis of SZ with no psychiatric diagnosis other than SZ on Axis I of the DSM-IV, decompensate organic disease, and mental retardation. The severity of symptoms was measured by a clinician using the Positive and Negative Symptom Scale (PANSS; Kay, 1987). All patients were stable (i.e., no need for hospitalization at inclusion and no major change in the patients' condition for 2 months prior to inclusion) and on antipsychotic medication with the following doses, expressed as mean and standard deviation: Haloperidol (*n* = 1) 100 mg; amisulpride (*n* = 1) 400 mg; olanzapine (*n* = 1) 300 mg; clozapine (*n* = 2) 550 mg (*SD* = 212); aripiprazole (*n* = 4) 267 mg (*SD* = 94). One patient received polypharmacy with risperidone and quetiapine (1156 mg). Some of them also received antidepressants and anxiolytics. The mean disease duration of patients was 14.2 years (*SD* = 9.9). The patients' mean age at the time of assessment was 36.3 (*SD* = 11.9) and the mean years of education amounted to 12.1 (*SD* = 1.6). The patients showed moderate severity of symptoms, with a total PANSS score of 63 (*SD* = 21.3) and sub-scores of 13.1 (*SD* = 4.3), 17.3 (*SD* = 7.0), and 32.6 (*SD* = 9.8), respectively, for positive, negative, and general psychopathology factors. The control group consisted of healthy volunteers recruited in the local community. They had no current or previous history of a major psychiatric disorder. The SZ and control groups did not differ significantly with regard to age [*t*_(18)_ = 2.6, *p* > 0.05] and education level [*t*_(18)_ = −1.5, *p* > 0.05]. The demographic and clinical data are summarized in Table 1. To give an overview of SZ patients' basic cognitive competence, information regarding patients' working memory and executive functions are presented as Supplementary Materials.

**Table 1:** **Demographic and clinical data on individuals with schizophrenia and healthy control participants**.

	**Schizophrenia**		**Healthy control**		
	**Mean**	***SD***	**Mean**	***SD***	***p*-value**
Age	36.3	11.9	33.7	12.5	0.641
Education level	12.1	1.6	13.6	1.8	0.067
**GENDER (MALE/FEMALE)**					
Duration of illness	14.2	9.9			
PANSS (positive)	13.1	5			
PANSS (negative)	17.3	7			
PANSS (general)	32.6	9.8			
PANSS (total)	63	21.3			

All participants were native French-speakers with no previous neurological history. Written consent forms were obtained from all participants, according to ethics guidelines set out by Aix-Marseille University and in accordance with the Declaration of Helsinki.

### Materials

Participants were tested individually using two tasks. First, we used the Hinting task (Corcoran et al., 1995) to assess participants' ToM ability. During this test, participants had to infer the intention behind disguised speech acts. The test included 10 brief stories involving two characters in which one of the protagonists drops a fairly clear hint. After the experimenter read the stories out aloud (as many times as necessary), the subject was asked to assess what the main protagonist in the story intended to say. If the subject did not make an inference or drew and inappropriate conclusion, more information was added to the story in the form of an even more obvious hint. The task had a maximum score of 20. An example of a stimulus is presented in Appendix.

Participants then performed an interactive task involving cooperation between a director (the participant) and a follower (the experimenter). As in traditional Mapstasks (see for instance Brown et al., 1983; Anderson et al., 1991), the director had to transfer a given route from his/her map to his/her follower's own as accurately as possible. The landmarks on the map were chosen so that the researchers could control the words produced by the participants during the game (e.g., by manipulating the phonetic characteristic of the landmarks) while ensuring that these words were produced in an interactional situation that is close to natural conversation. The “naturalness” of such corpora is shown by the disfluencies and hesitations produced by the two partners during the task. We recorded both the director's and follower's productions, but we analyzed only the director's productions. The follower's continual feedback also proves that common ground was being established between the two interactional partners.

**Figure 1:**
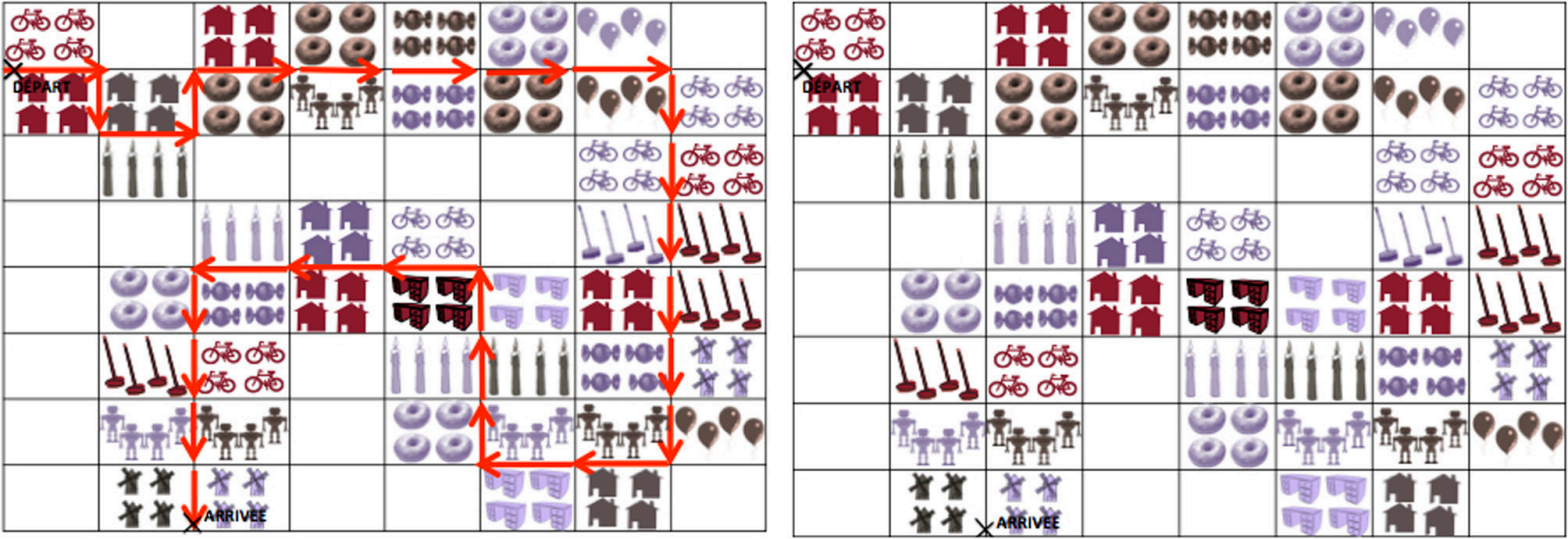
**Maps used for the experiment; the director's map includes the route (left panel) while the follower's map does not include it (right panel)**.

In this task, the director had to indicate 20 critical pairs of landmarks to the follower (e.g., *Tu dois passer entre les BOUGIES violettes et les BONBONS violets* “You have to pass between the purple candles and the purple candies”). Each pair of landmarks was composed of two noun-adjective fragments; the noun in the second fragment could be either identical to the noun in the first (e.g., *BONBONS marron* “brown candies” vs. *BONBONS violets* “purple candies”) or could contrast with it (e.g., *BOUGIES violettes* “purple candles” vs. *BONBONS violets* “purple candies”). This interactive task enabled us to directly measure the acoustic correlates of AP prosodic phrasing (i.e., f0 values and duration properties) of the same target noun that could appear in two pragmatic contexts depending on its discourse status (given vs. contrastive). Since our aim was to examine the prosodic phrasing of the Accentual Phrase through both duration and melodic analyses, the names of the landmarks contained only sonorants in order to facilitate the analysis of the pitch curve. We also controlled for the number of syllables within Accentual Phrases, which has been shown to affect prosodic phrasing in French (Astésano, 2001; Welby, 2006; Michelas and D'Imperio, 2012). In addition to the 20 critical pairs of landmarks, 7 noun-adjective pairs of fillers were added. Each map included 32 pairs of landmarks. Figure 1 shows the two maps used for the experiment.

### Procedure

In our study director and follower were seated, facing each other, separated by an opaque screen. They both had the same map but only the director knew the route. They could not see each other's maps. Both maps had the same points of departure and arrival.

Before the experimental phase (i.e., the interactive task), directors performed a familiarization phase during which they had to report aloud what they saw described in pictures. This phase, which took 2 min to complete, included 30 pictures with all possible combinations of shapes and colors presented in a random order by the director. The purpose was to ensure that directors could identify and use a consistent label for each target object and color. They were asked to remember these labels, as they would see the same pictures in the next phase of the game.

During the experimental phase, participants were asked to use the same labels as in the familiarization phase. The experimenter gave instructions to the participant as follows: We are going to take part in an interactive game. The aim of this game is to transfer as accurately as possible a given route from one map to the other map. During the game, you will see the route on your map but I won't see it on my own map. Note that we both have the same departure and arrival points. At each intersection, you have to show me the route to follow by indicating to me the pair of landmarks between which I have to pass (e.g., *Tu dois passer entre les BOUGIES violettes et les BONBONS violets* “You have to pass between the purple candles and the purple candies”). You cannot indicate only one set of landmarks; you must mention both sets of objects. As you did during the first part of the experiment, you have to indicate each set of objects by mentioning properties of both their shape and color. Note that the sets of objects on the map correspond to the objects you saw during the previous part of the experiment. If I get lost during the game, I will stop you and ask you to help me to find the route. Do not hesitate to check whether I am on the right way at any time during the game. Are you ready?'

Directors and followers were recorded in a quiet room using a Zoom H4N Handy Recorder and a Headset Cardioid Condenser Microphone (AKG C520). Recordings were saved as .wav files at a 44.1-kHz sampling rate with 16 bit resolution. Directors all performed the Hinting task before the interactive task.

### Hypothesis and predictions

Because both partners knew that at each intersection the route to follow passes between a first set and a second set of objects in our experimental design, they had to pay attention to the similarities/differences between objects in two-by-two comparisons. Crucially, at each intersection (i.e., each pair of objects), the director had to indicate to his/her follower whether the second noun was identical relative to the first one (*BONBONS* “candies” vs. *BONBONS* “candies”) or whether it contrasted with it (*BOUGIES* “candles” vs. *BONBONS* “candies”). To do so, the speaker had to: (i) share with his/her partner a set of alternatives appropriate for the immediately ensuing discourse and (ii) prosodically mark the contrastive status of one of the possible alternatives shared with the listener. We predicted that HC speakers who were able to take the listener's standpoint - and thus to participate in the construction of the common ground—would use prosodic phrasing to indicate the given vs. contrastive status of target nouns. Specifically, we expected HC speakers to produce the target noun within the same Accentual Phrase (AP) as the following adjective in the given condition (i.e., *[Les bonbons violets]*^AP^ “The purple candies”) while they would produce the target nouns separately from the following adjective in the contrastive condition (i.e., *[Les bonbon*s]^AP^ *[violets]*^AP^). However, because of their ToM impairment, we expected SZ participants to have difficulties in constructing shared knowledge with their interlocutor about the set of alternatives appropriate for the immediately ensuing discourse. As a consequence, they would be unable to prosodically mark the informational status of one of these alternatives as HC participants did. Thus, we expected SZ participants to produce the two types of phrasing exhibited by HC participants (either one AP or two APs) regardless of the discourse status of referents.

### Measures and acoustic annotation

**Figure 2:**
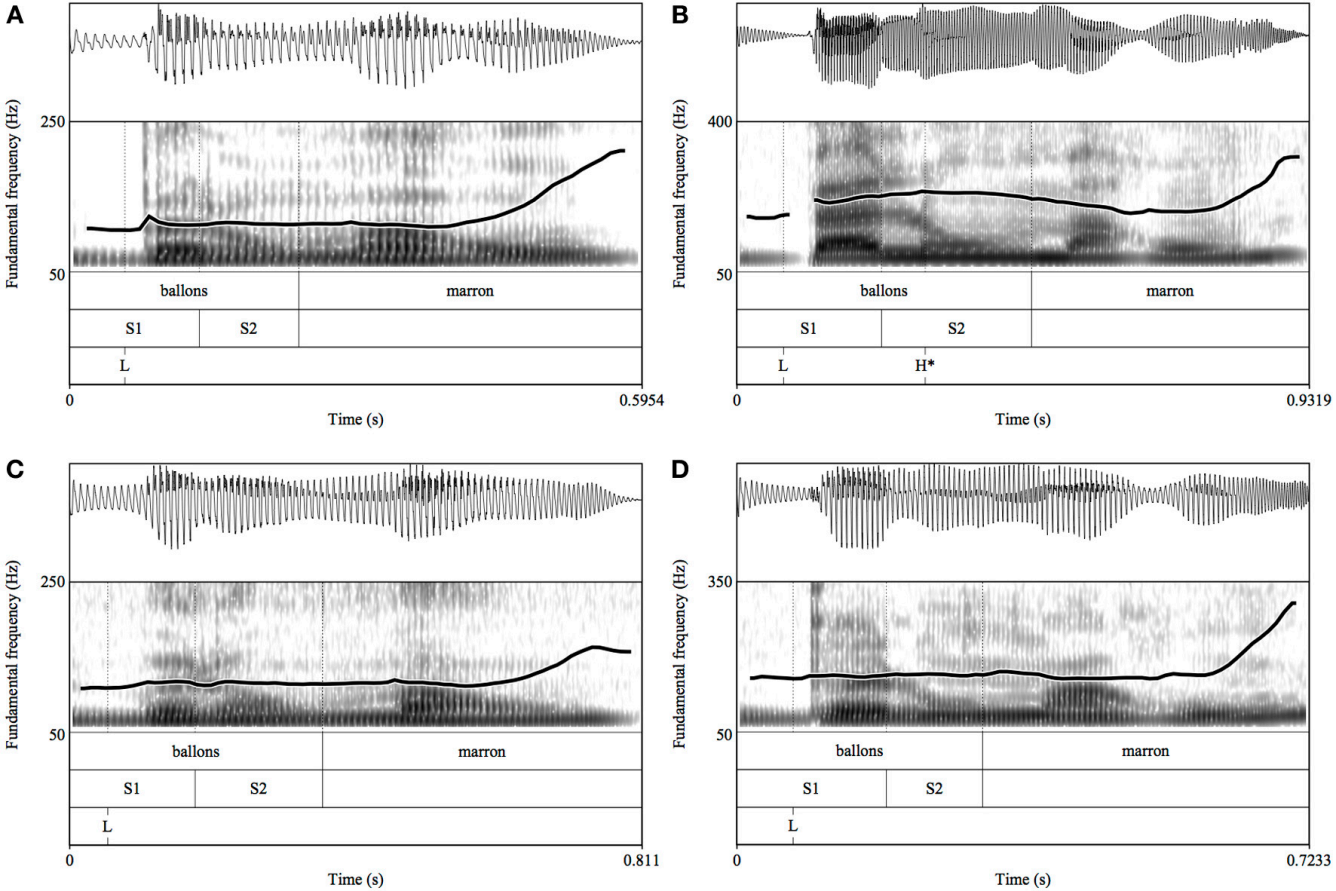
**Illustration of the annotation of two noun-adjective pairs produced either by a HC speaker in the given condition (A) and in the contrastive condition (B) or by a SZ speaker in the given condition (C) and in the contrastive condition (D).** The noun in **(B)** is phrased in a separate AP from the adjective (as noted by the LH^*^ notation) while all three fragments were parsed as a single AP. The gloss is: the brown balloons.

The noun-adjective pairs containing neither disfluencies/hesitations nor object appellation errors were analyzed for a total of 323 noun-adjective pairs (80.75% of the original data). Following previous studies on French intonation (see Pasdeloup, 1990; Jun and Fougeron, 2000, 2002; Astésano, 2001; Welby, 2006; Michelas and D'Imperio, 2012, *among others*), we used pre-boundary lengthening and pitch contours as the two main correlates of AP prosodic phrasing. As a consequence, the first and last syllable of target nouns were automatically segmented by means of Easyalign (Goldman, 2007) and manually tagged S1 and S2, respectively (see Figure 2 below). Minimum and maximum f0 values associated with the last syllable of the target nouns (or just beyond it) were also automatically placed using Praat scripts (Boersma and Weenink, 2009) and hand-corrected by the first author of this paper to check for octave errors (labeled L and H respectively). Figure 2 shows an illustration of the labeling conducted for two noun-adjective pairs.

Based on these tagged features, the first author annotated the AP prosodic phrasing of noun-adjective pairs. Following Jun and Fougeron's definition of the French AP (Jun and Fougeron, 1995, 2000, 2002; see also Welby, 2006; Michelas and D'Imperio, 2012), the target noun was considered as phrased in a separate AP from the following adjective when a pitch accent was associated with its last syllable. We used two specific criteria to define the presence of an AP right boundary. First, we considered that a pitch accent was actually produced by the speaker if the maximum hertz value of the target syllable (S2) was at least 10% higher than that of the preceding early L following the acoustic criterion used in Astésano (2001) for the pitch accent detection. We also verified that the syllable was lengthened. To do so, we checked whether the duration of S2 was at least 10% longer than that of S1 (see Figure 2). This is in line with Michelas and D'Imperio (2012) who demonstrated that an AP-final syllable is on average 10% longer than a syllable contained within an AP.

## Statistical analysis and results

The analyses were performed as follows. We first performed an unpaired *t*-test on the hinting task to evaluate participants' ToM ability. Second, we performed a mixed logit model (Baayen, 2008) on the AP prosodic phrasing produced by participants to determine whether target nouns were parsed within the same AP as the following adjectives or as separate prosodic units. To confirm the reliability of the acoustic criteria used to indicate the presence or absence of an AP right boundary on the last syllable of the target nouns, we also performed two linear mixed models on the two main acoustic correlates of the AP right boundary (i.e., duration of the AP-final syllable and f0 values associated with the final f0 peak). We calculated participants' scores in prosodic phrasing by measuring the deviance from the expected prosodic phrasing. We then used Spearman correlation tests to assess (i) a possible relationship between ToM and other disease-related attributes (i.e., duration of illness, symptoms and medication effects) and (ii) a possible relationship between ToM and executive functions in the SZ group. Finally, a Spearman correlation test allowed us to investigate the relationship between the ToM score in the hinting task and the prosodic phrasing score in the interactive task.

### ToM score of participants in the hinting task

An unpaired *t*-test performed on the hinting task revealed that SZ participants performed significantly worse than HC participants in this task, *t*_(18)_ = −3.68, *p* > 0.002 (SZ: mean = 14.9, *SD* = 2.1; HC: mean = 17.9, *SD* = 1.5).

### Prosodic phrasing produced by participants in the interactive task

**Figure 3:**
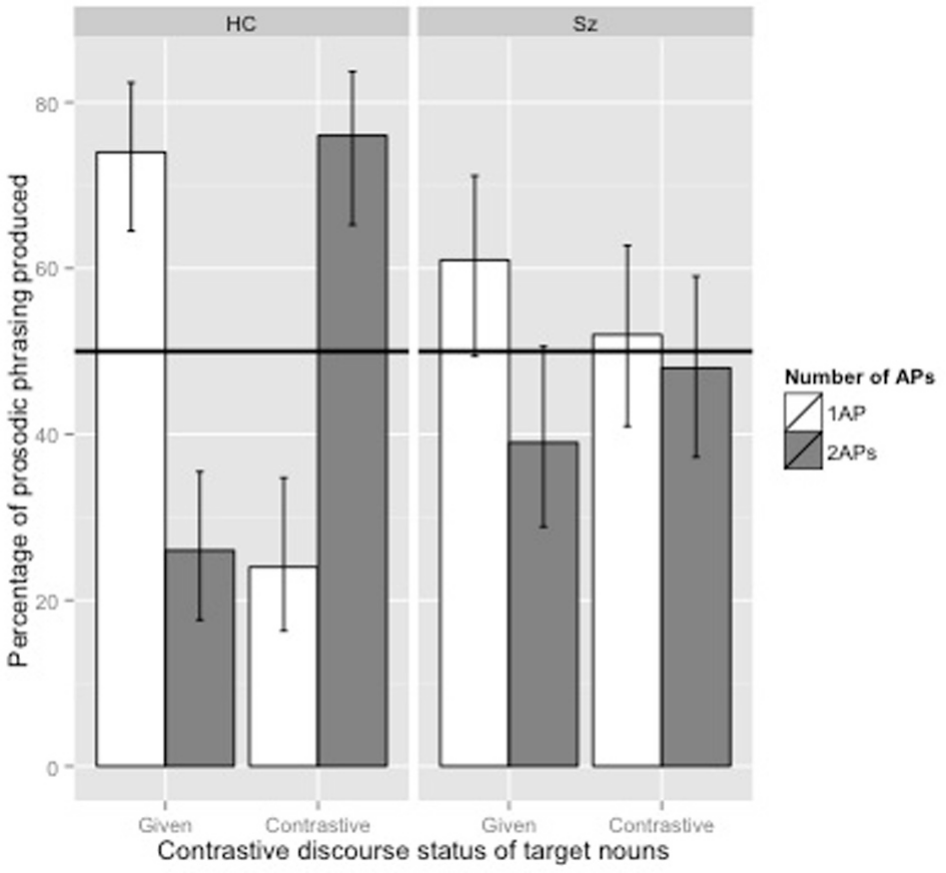
**Percentage of prosodic phrasing produced by healthy controls participants (HC) and participants with schizophrenia (SZ) depending on the number of APs they produced (1 AP vs. 2 APs) and the contrastive discourse status of target nouns (given vs. contrastive).** Error bars show a default 95% confidence interval.

The percentage of AP prosodic phrasing depending on whether the noun was parsed as a separate AP (2APs) or within the same AP as the adjective (1AP) for HC and SZ is shown in Figure 3.

To test the statistical relevance of the prosodic phrasing produced by participants, we employed mixed effects logistic regression modeling. We used lme4 (Bates et al., 2008) and languageR packages (Baayen, 2008) as described in Baayen (2008) implemented in R (R Development Core Team, 2008). Our binary dependent variable was the prosodic phrasing produced by participants (1AP = 1, 2APs = 0). We first tested a model including the contrastive discourse status of target nouns (DiscourseStatus), the group of participants (Group) and their interaction as fixed factors. The random part of the model included random intercepts for participants and items and random slopes allowing for the effects of the predictors to differ across participants or items for all between-unit predictors (see for instance Baayen and Milin, 2010; Barr et al., 2013): APnumber~ DiscourseStatus ^*^ Group + (1 + DiscourseStatus | participant) + (1 + DiscourseStatus^*^Group | item). The model was then simplified by excluding the slopes since their inclusion did not make the model fit the data better. Note that the Akaike Information Criterion (AIC) score of the retained model (AIC = 388.1) was lower than that of the model with the maximum random effect structure (AIC = 404.7). Thus, the retained model included DiscourseStatus and Group and their interaction as fixed factors, and participant and item intercepts only as random factors: APnumber~ DiscStatus^*^Group + (1|participant) + (1|item). This model included 323 observations. Results of the mixed effects logistic regression are given in Table 2.

**Table 2:** **Results of the mixed effects logistic regression fitted on the AP prosodic phrasing produced by participants**.

**RANDOM EFFECTS**				
	**Name**	**Variance**	**Std. Dev**	
Participant	Intercept	0.79490	0.89157	
Item	Intercept	0.35673	0.59727	
**FIXED EFFECTS**				
	**Coefficient estimate**	**Standard error**	***z***	***p***
(Intercept)	−1.2384	0.4291	−2.886	< 0.01
DiscourseStatus (Contrastive)	2.5982	0.3917	6.663	< 0.01
Group(SZ)	0.5948	0.5470	1.087	0.276890
Contrastive:SZ	−2.0395	0.5325	−3.830	< 0.01

In line with our hypothesis, the model showed that HC participants produced more 2APs prosodic phrasings when the target noun was contrastive than when it was given (*z* = 6.633, *p* < 0.01, see Table 2). In addition, the interaction between the Discourse Status and the Group was significant (*z* = −3.830, *p* < 0.001, see Table 2). The results were the same in the model with the maximum random effect structure [effect of DiscourseStatus(Contrastive): *z* = 5.176; effect of Group(SZ): *z* = 0.866; effect of the interaction (Contrastive:SZ): *z* = −2.876]. In order to better interpret the interaction between the DiscourseStatus and the Group, we used two separate logit mixed models for HC participants and for SZ participants. As for the preceding mixed logit model, the retained model included DiscourseStatus as fixed factor and participant and item intercepts only as random factors. The results confirmed that the effect of DiscourseStatus was significant for the HC group (β = 2.5358, *SE* = 0.3833, *z* = 6.616, *p* < 0.01) but not for the SZ group (β = 0.6335, *SE* = 0.3740, *z* = 1.694, *p* = 0.0902). Note that the results were the same in the two models with the maximum random effect structure (for the HC group: *z* = 5.2; for the SZ group: *z* = 1.7).

**Figure 4:**
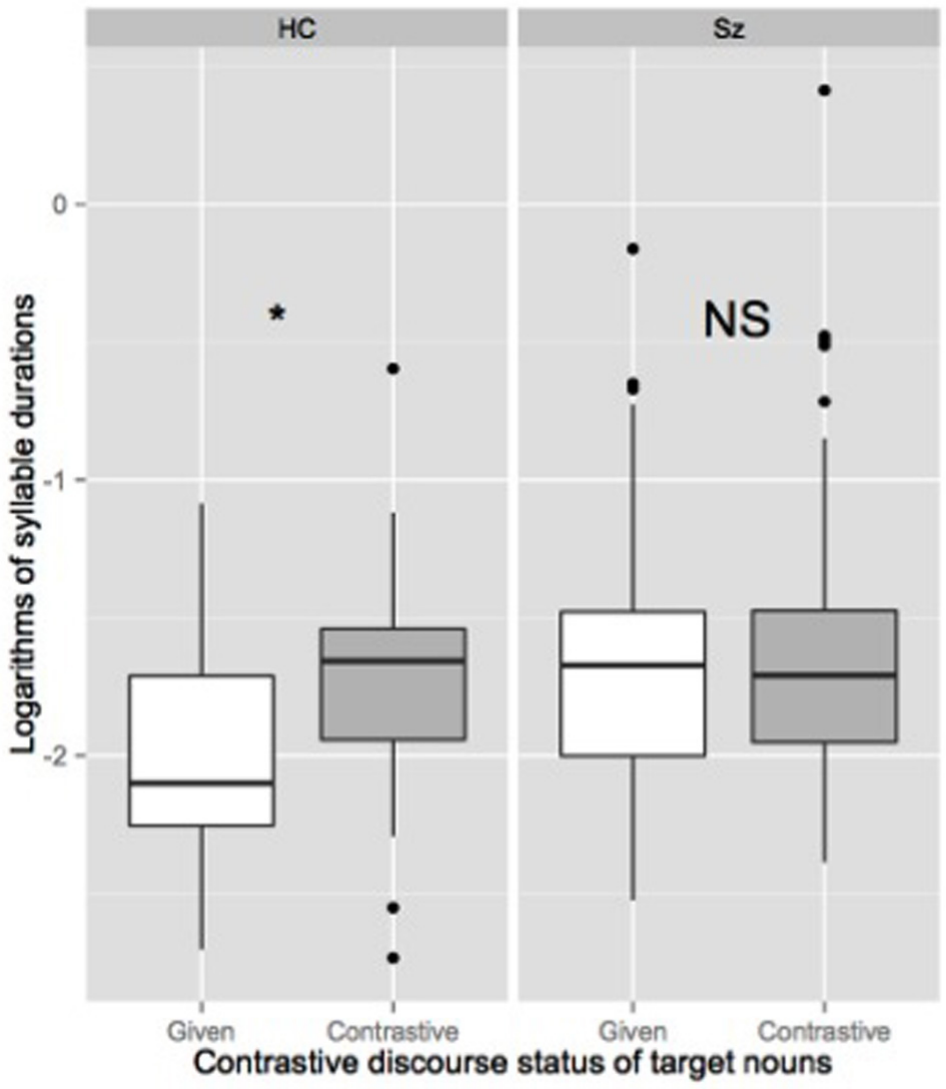
**Boxplot of logarithms of duration of target nouns' last syllable produced by healthy controls participants (HC) and participants with schizophrenia (SZ) depending on their contrastive discourse (given vs. contrastive).** The spacing between the different parts of the box indicates the degree of dispersion in the data. The bottom and top of the box represent the first and third quartiles, and the band inside the box corresponds to the second quartile (the median). Whiskers indicate the variability outside the upper and lower quartiles. Outliers are plotted as individual points. ^*^Significance level is set to < 0.05.

**Figure 5:**
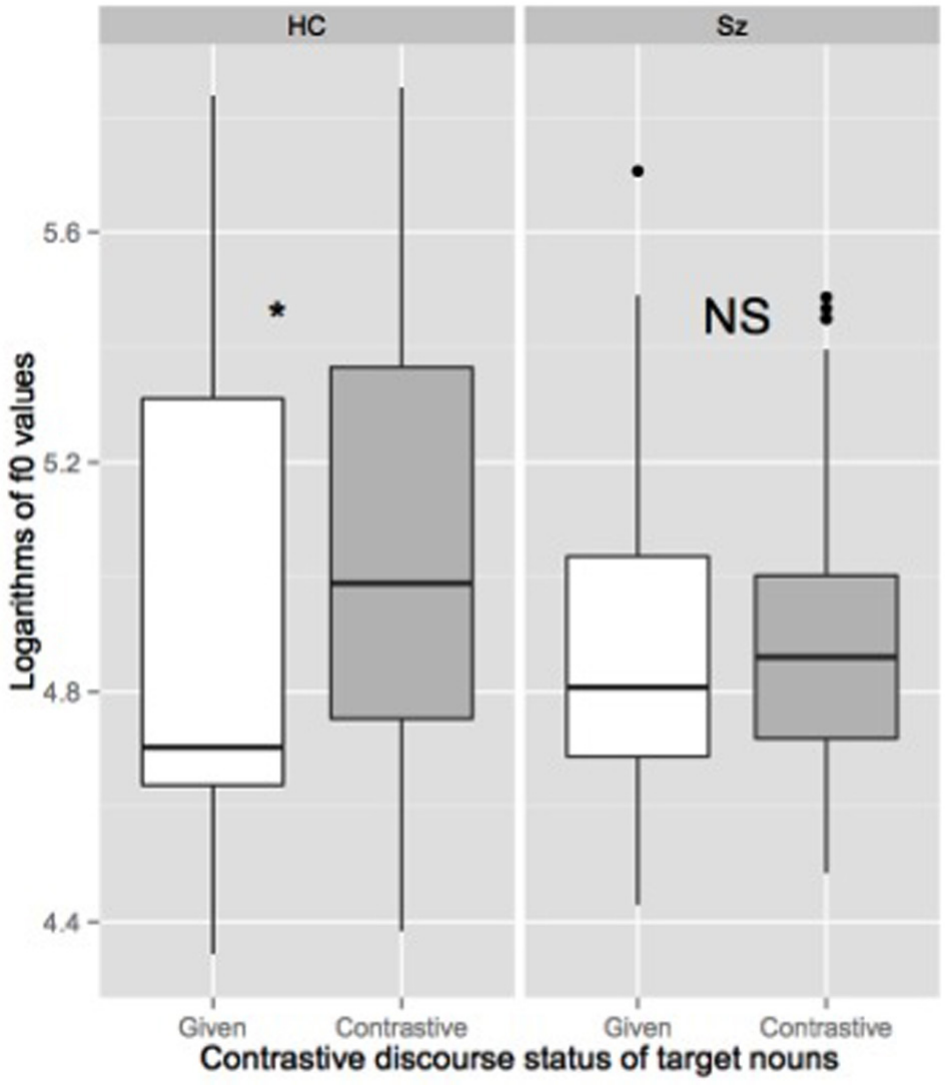
**Boxplot of logarithms of f0 maxima associated to the last syllables of target nouns produced by healthy controls participants (HC) and participants with schizophrenia (SZ) depending on their contrastive discourse status (given vs. contrastive).** ^*^Significance level is set to < 0.05.

We also fitted two linear mixed effects models on the two acoustic parameters we used to define the presence of an AP right prosodic boundary. In order to normalize the variability found both within and across speakers, both duration and f0 measures were log transformed. Thus, our two linear dependent variables were logarithms of duration of the last syllables of target nouns and logarithms of values of the f0 maxima of target nouns. Our two binary explanatory variables were the contrastive discourse status of target nouns (given vs. contrastive) and the group of participants (HC vs. SZ). As for the first mixed logit model, we first tested two linear mixed models including the contrastive discourse status of target nouns (DiscourseStatus) and the group of participants (Group) and their interaction as fixed factors. The random part of the models included random intercepts for participants and items and random slopes allowing for the effects of the predictors to differ across participants or items for all between-unit predictors. Following the same procedure, the two models were then simplified by excluding the slopes since their inclusion did not make the model fit the data better. Note that in all analyses, the results were the same in the retained models than in the models with the maximum random effect structure. The two retained models included 323 observations each. *p*-values were estimated using the Markov Chain Monte Carlo (MCMC). In line with our hypothesis, the last syllable of the target nouns was longer in the contrastive condition than in the given condition for HC (*t* = 3.898, pMCMC < 0.01). The effect of the Group was significant while the interaction between the DiscourseStatus and the Group was not significant (see Table 3 and Figure 4). Two separate linear mixed models (including DiscourseStatus as fixed factor and participant and item intercepts only as random factors) confirmed that the effect of Discourse status on the logarithms of duration of the last syllable of target nouns was significant for the HC group (β = 0.22090, *SE* = 0.04901, *t* = 4.507, pMCMC < 0.01) but not for the SZ group (β = 0.08122, *SE* = 0.06907, *t* = 1.176, pMCMC = 0.2632). Similarly, maximum f0 values associated with the last syllable of the target nouns were higher in the contrastive condition than in the given condition for HC (*t* = 7.58, pMCMC < 0.01). The effect of the Group was not significant while the interaction between the DiscourseStatus and the Group was significant (see Table 3 and see Figure 5). Two separate linear mixed effects models (including DiscourseStatus as fixed factor and participant and item intercepts only as random factors) confirmed that the effect of DiscourseStatus on logarithms of f0 values of the last syllable of target nouns was significant for the HC group (β = 0.1570, *SE* = 0.0226, *t* = 6.95, pMCMC < 0.01) but not for the SZ group (β = 0.0258, *SE* = 0.01969, *t* = 1.31, pMCMC = 0.2708).

**Table 3:** **Results of the linear mixed models fitted on the logarithms of duration values and on the logarithms of f0 values**.

	**Coefficient estimate**	**Standard error**	***t***	**pMCMC**
**FIXED EFFECT ON THE LOGARITHMS OF DURATION VALUES**				
(Intercept)	−1.9609	0.08234	−23.816	< 0.01
DiscourseStatus(Contrastive)	0.21869	0.05611	3.898	< 0.01
Group(SZ)	0.24915	0.10622	2.346	< 0.05
Contrastive:SZ	−0.137	0.082	−1.671	0.0920
**FIXED EFFECT ON THE LOGARITHMS OF F0 VALUES**				
(Intercept)	4.89564	0.09750	50.21	< 0.01
DiscourseStatus(Contrastive)	0.15740	0.02078	7.58	< 0.01
Group(SZ)	−0.02214	0.022	−0.99	0.4236
Contrastive:SZ	−0.1351	0.03056	−4.43	< 0.01

### Relationship between the interactive task and the hinting task

**Figure 6:**
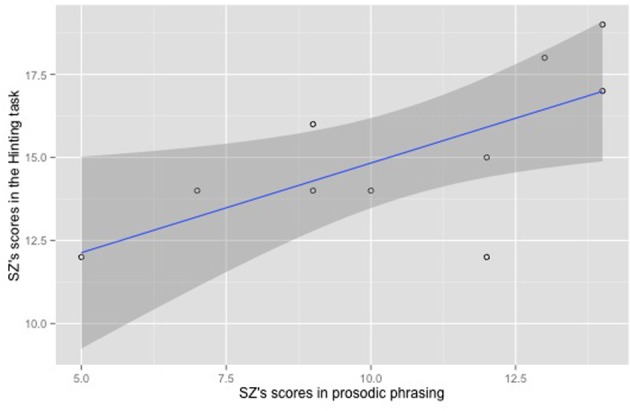
**Scatterplot illustrating SZ participants' scores in prosodic phrasing vs. their scores in the hinting task**.

To determine whether the prosodic phrasing produced by SZ participants was correlated with their ability to take into account the listener's knowledge, we tested the relationship between the prosodic phrasing they produced in the interactive task and their ToM score in the hinting task. We first calculated SZ participants' ability to use prosodic phrasing as an encoder of contrastive status of discourse referents. To do so, we measured the deviation between the prosodic phrasing they produced and the prosodic phrasing we expected. Let us take an example. Participant P29 produced 9 noun-adjective pairs in the given condition and 9 noun-adjective pairs in the contrastive condition without any disfluencies or hesitations. This participant grouped the noun and the color adjective in the same Accentual Phrase 3 times (3 1AP-phrasing) while he produced 6 times the target noun as a separate AP from the adjective (6 2AP-phrasing) in the given condition. In addition, he also grouped the noun and the color adjective in the same Accentual Phrase 3 times (3 1AP-phrasing) while he produced 6 times the target noun as a separate AP from the adjective (6 2AP-phrasing) in the contrastive condition. Since we expected all noun-adjective pairs in the given condition to be produced as a single AP (i.e., 9 1AP-phrasing for P29), and all noun-adjective pairs in the contrastive condition to be produced as two APs (i.e., 9 2AP-phrasing for P29), participant P29 received a 10/20 prosodic phrasing score in the interactive task (i.e., 3/9 in the given condition + 6/9 in the contrastive condition = 9/18 = 10/20). Note that because the hinting task has a maximum score of 20, we use a 0–20 scale to evaluate prosodic phrasing. A spearman correlation test showed that SZ participants' scores in prosodic phrasing were correlated with their scores in the hinting task (*r* = 0.711, *p* < 0.05). The scatterplot of SZ participants' scores in prosodic phrasing in the interactive task vs. their scores in the hinting task is given in Figure 6.

### Relationship between ToM performances and clinical variables

To assess the relationship between ToM and other disease-related attributes such as duration of illness, symptoms and medication effects, a Spearman's correlation analysis was conducted in the SZ group. No correlation was found between ToM assessed by the Hinting task and the duration of illness (*r* = −0.158, *p* > 0.05), the symptoms (PANSS positive: *r* = 0.009, *p* > 0.05; PANSS negative: *r* = −0.018, *p* > 0.05; PANSS general: *r* = −0.203, *p* > 0.05; PANSS total: *r* = −0.068, *p* > 0.05) and the medication in chlorpromazine equivalent (*r* = 0.263, *p* > 0.05). These correlation analyses were also performed with prosodic phrasing score. They also lead to an absence of correlation between the prosodic phrasing score and the duration of illness (*r* = 0.145, *p* > 0.05), the symptoms (PANSS positive: *r* = −0.47, *p* > 0.05; PANSS negative: *r* = −0.092, *p* > 0.05; PANSS general: *r* = −0.208, *p* > 0.05; PANSS total: *r* = −0.104, *p* > 0.05) and the medication in chlorpromazine equivalent (*r* = 0.0345, *p* > 0.05). Results of Spearman's correlation analyses between ToM performances and neuropsychological variables are presented as Supplementary Materials.

## Discussion

This study aimed at determining whether SZ individuals who experienced ToM impairments used linguistic prosody to encode contrastive status of discourse referents as HC did. To this end, we used an interactive task to elicit pairs of noun-adjective fragments in which the noun in the second fragment could contrast with the noun in the first fragment or not. This task allowed us to measure the prosodic phrasing produced by participants (in one or two APs) through two acoustic measures (i.e., f0 and duration values) associated with the last syllable of target nouns. The main result showed that unlike HC participants, SZ participants did not use AP prosodic phrasing to encode the given vs. contrastive status of discourse referents in an appropriate way in connection with their difficulties in attributing knowledge to the listener.

Focusing on the interactive task, we found that HC participants preferentially produced a single AP when the target noun was given (e.g., *tu passes entre les BONBONS marron et [les BONBONS violets]^AP^* “*You have to go between the brown candies and the purple candies*”), while they preferentially produced two APs when the target noun contrasted with the noun of the first noun-adjective fragment (e.g., *tu passes entre les BOUGIES violettes et [les BONBONS]^AP^ [violets]^AP^* “*You have to go between the purple candles and the purple candies*”). Thus, HC speakers rephrased the target noun as a separate AP from the adjective to warn their interlocutor that it constituted a contrastive entity relative to the noun of the preceding noun-adjective fragment (see Figure 2). Consequently, we found that the last syllables of target nouns produced by HC speakers were longer and associated with higher f0 values when the noun had a contrastive status than when it had a given status. This is in line with previous studies showing that the contrastive information tended to form its own AP in French (Féry, 2001; Dohen and Loevenbruck, 2004; Beyssade et al., 2009; Chen and Destruel, 2010).

However, we found that SZ participants did not produce more 1AP prosodic phrasing than 2AP prosodic phrasing when the target noun was given (see Figure 2). Similarly, they did not prefer a 2AP prosodic phrasing to a 1AP prosodic phrasing when the target noun was contrastive. This shows that while SZ participants were able to produce both types of prosodic phrasing, they did not use it to encode the contrastive status of referents. Rather, they produced either one or two APs regardless of the discourse status of target nouns. As a consequence, the last syllable of the target noun was neither longer nor higher in given contexts than in contrastive contexts in SZ participants' speech. These results suggest that SZ participants did not use AP prosodic phrasing in an appropriate way to encode discourse status. Moreover, SZ's impairment in attributing knowledge to their interlocutor was confirmed by their performances in a classical ToM task in which they performed more poorly than HC participants.

Focusing on the relationship between the interactive task and the hinting task, we found that the scores of SZ participants in the hinting task correlate with their scores in prosodic phrasing. This suggests that the abilities of SZ participants to use prosodic phrasing to encode the contrastive status of discourse referents would decrease with their ability to attribute knowledge to others. Thus, the correlation we found between SZ's participants score in the hinting task and their score in prosodic phrasing suggest that their use of prosody of contrast could be linked to their ToM impairments. In fact, in the interactive paradigm we used, the director (the participant) had to tell the follower how to chart the route onto the follower's map as accurately as possible. As the conversation progressed, the speaker had to structure the information into two parts, the background and the contrastive information. To do so, the speaker may construct mutual knowledge with his/her interlocutor about a set of appropriate alternatives. Specifically, to indicate to the listener which alternative was the contrastive one, the speaker shared with his/her listener a set of possible alternatives. Our SZ participants might be unable to establish this set of alternatives and thus unable to use prosody of contrast in an appropriate way because the way speakers refer to shared knowledge is typically affected in ToM impairments. In addition, the absence of correlation between clinical (i.e., duration of illness, symptoms, medication) or neuropsychological variables and the score on the hinting task or the prosodic phrasing score gives support to the fact that the correlation between ToM and prosody would be really about ToM and not about some other measure that covaries with ToM. Overall, these results appear to support the idea that linguistic prosody could represent a relevant marker of ToM abilities in interactive situations and that the interactive task we used would be a good paradigm for studying ToM ability during conversation. Interestingly, our results are in line with those of Champagne-Lavau et al. (2009) who reported SZ participants' impairments in ToM during conversation. This study showed that, during conversation, the inappropriate use of reference markers (i.e., indefinite articles to mark old information and definite articles for new information) by participants with SZ reflected their difficulties in taking into account the information they shared with their interlocutor and in correctly attributing intentions, knowledge and beliefs to their interlocutor. This study relied on the fact that linguistic markers of referential cohesion, such as indefinite (*a* mountain) or definite (*the* mountain) descriptions, play a primary role in the construction of shared knowledge between interlocutors (Pickering and Garrod, 2004). Thus, linguistic markers of referential cohesion such as indefinite/definite descriptions reflected ToM ability in natural conversation situations. In line with the idea of studying several components of the ToM ability in social interactions, our results suggest that the use of linguistic prosody as a potential additional linguistic marker of ToM.

However, this study has some limitations. First, although robust and consistent differences were found across groups in the use of f0 and duration to encode AP prosodic phrasing, our results are preliminary. A larger sample of participants in the subgroup of SZ patients is required to confirm the positive correlation found between our interactive task and our ToM task. Given that our SZ patients were mostly middle-aged males, mildly affected and ill for more than 5 years, a more diverse group of patients would also be preferable to make our sample more representative of patients with SZ. Second, concerning how our SZ participants performed the experimental phase, one could argue that the interactive paradigm we used was more difficult to achieve for SZ participants than for HC. In fact, there are two possible scenarios. First, it could be possible that the difference we found in the prosodic encoding of discourse status between HC and SZ would reflect the SZ's difficulties in constructing shared knowledge with their interlocutor. Second, it could also be due to the fact that the more difficult a production task is in a general sense, the less likely one is to produce prosodically informative contrasts spontaneously. Following this idea, our SZ participants would use prosodic phrasing differently from HC participants because the interactive paradigm we used would be a more burdensome or a more demanding task situation for them than for HC. We favor the first alternative for three reasons. First, it is important to note that in 100% of cases, our SZ participants successfully completed the interactive task. Specifically, all our participants (including both SZ and HC participants) succeeded in guiding the experimenter from the point of departure to the point of arrival. Second, SZ participants were able to use both types of prosodic phrasing (either 1 AP or 2 APs), just as HC participants did. Thus, combined with the absence of correlation between clinical (i.e., duration of illness, symptoms, medication) or neuropsychological variables and the score of prosodic phrasing, this provides additional evidence that our SZ participants did not suffer from difficulties in speech articulation. Rather, they experienced difficulties in attributing knowledge to their interlocutor, a capacity that may be crucial to prosodically encode pragmatic functions such as the marking of contrastive statuses of discourse referents. Finally, the total time and the total number of speaking turns (defined as series of speech with no intervention by the partner) required to perform the task are objective measures (Feyereisen et al., 2007; Champagne-Lavau et al., 2009) that may tease apart the two alternatives. Results of Student *t*-tests revealed no significant difference between SZ patients and HC participants on the total time [*t*_(18)_ = −1.66, *p* = 0.11; mean for SZ: 208.8, SD for SZ: 63.46; mean for HC: 169.10; SD for HC: 41.01] and on the total number of speaking turns [*t*_(18)_ = −0.86, *p* = 0.40; mean for SZ: 31.5; SD for HZ: 4.6; mean for HC: 30.0, SD for HC: 2.91]. Despite the fact that (i) the total time speaking and the total number of speaking turns tend to be slightly greater for SZ participants than for HC and (ii) a larger sample of participants is required to confirm that these two objective measures do differ between SZ and HC participants in a more general manner, this supports the interpretation that SZ participants managed to understand and carry out the task in an appropriate way. Thus, the source of the difference we found on the prosodic encoding of contrasts between SZ and HC appears to reflect participants' ToM ability rather than difficulties in achieving the task.

Finally, a possible third limitation is that the paradigm we used allowed us to measure the acoustic properties of discourse referents in initial position in the fragment (e.g., {les BOUGIES violettes}^1st fragment^ {et les BONBONS violets}^2nd fragment^) but not in final position in the fragment (e.g., {les BONBONS marron}^1st fragment^ {et les BONBONS violets}^2nd fragment^). Since the last syllable of the adjectives always coincided with the last syllable of the sentences in our study, the acoustic properties encoding AP right boundaries were superimposed on the acoustic properties encoding the end of the sentences. For this reason, it is impossible to factor out the function of prosodic phrasing in the discourse from its demarcative function when in final position in the fragment. It would be interesting for future work to vary the position of the target word in the sentence in order to include it as a variable in the experimental paradigm. To do so, it would be suitable to introduce additional material after the color adjective by inducing more complex noun phrases (e.g., *les bonbons violets de la petite fille* “the purple candies of the little girl”) rather than simple noun-adjective fragments like the ones we used. In addition to prosodic restructuring phenomena, previous research on French intonation has given evidence for additional markers of the contrastive vs. given status of discourse referents, such as the presence of an initial f0 rise near the beginning of the AP (Beyssade et al., 2009; German and D'Imperio, 2010). Thus, it could also be interesting for further work to investigate to what extent other prosodic markers of contrastive status could be impaired in SZ speech.

In conclusion, this study showed that SZ participants did not use prosodic phrasing to encode the contrastive status of discourse referents as HC participants did. In addition, SZ participant's difficulties in using prosody of contrast were correlated to SZ' participants score in a classical ToM task (i.e., the hinting task). This suggests that SZ's production of linguistic prosody could reflect their difficulties in attributing knowledge to others during conversation. Given that our results are preliminary, more research is required to confirm the correlation we found between ToM ability and the use of prosody of contrasts. According to Brüne and Schaub (2012) some SZ patients do not show ToM impairments. Thus, the next step of this research will be to show that such patients without ToM difficulties would be able to produce prosody of contrasts as HC do. Moreover, we know that prosodic cues produced by speakers to mark discourse structure are processed by healthy listeners to interpret the contrastive status of discourse referents (Dahan et al., 2002; Ito and Speer, 2008). Thus, research must be further developed to determine whether SZ individuals would be able to retrieve prosodic cues to interpret the contrastive status of referents to more precisely determine the impact of ToM in the production and the processing of linguistic prosody.

### Conflict of interest statement

The authors declare that the research was conducted in the absence of any commercial or financial relationships that could be construed as a potential conflict of interest.

## Appendix

### Item extracted from the hinting task

Paul has to go to an interview and he is running late. While he is cleaning his shoes, he says to his wife, Jane: “I want to wear my blue shirt but it's very creased.”

Question: What does Paul really mean when he says this?

If necessary add: Paul goes on to say: “It's in the ironing basket.” Question: What does Paul want Jane to do?
