# Supplementary material for: Do patients with schizophrenia use prosody to encode contrastive discourse status?
Source: Front Psychol. 2014 Jul 18;5:755. doi: 10.3389/fpsyg.2014.00755 (PMC4102879; doi:10.3389/fpsyg.2014.00755)
Supplement: Supplementary file 1 [file DataSheet1.DOCX]

**Information regarding patients’ working memory and executive functions**

Working memory was assessed with the digit Span subtest from the WAIS (Wechsler, 1981). Executive functions were assessed with the letter fluency and the categorical fluency with the total number of words as shifting and word generation indexes (Cardebat et al., 1990), and with the Modified version of the Wisconsin Card Sorting Test with the total number of categories and the percentage of perseverative errors as shifting indexes (Nelson, 1976).

Table S1. Patients’ neuropsychological characteristics

|  | Mean | SD |
| --- | --- | --- |
| Digit span forward | 5.8 | 0.8 |
| Digit span backward | 4.4 | 1.5 |
| Letter Fluency | 21.1 | 3.4 |
| Categorical Fluency | 30.9 | 5.2 |
| WCST-Category | 4.7 | 1.6 |
| WCST-% of perseverative errors | 23.2 | 17.9 |

**Relationship between ToM performances and neuropsychological variables**

To assess a possible relationship between ToM and executive functions, a Spearman’s correlation analysis was conducted in the SZ group. No correlation was found between ToM assessed by the Hinting task and the digit span forward (r = 0.151, p > 0.05), the digit span backward (r = -0.115, p > 0.05), the letter fluency (r = -0.229, p > 0.05), the categorical fluency (r = 0.156, p > 0.05), the WCST-category (r = 0.239, p > 0.05) and the WCST-percentage of perseverative errors (r = 0.106, p > 0.05).
